# Use of rectal artesunate for severe malaria at the community level, Zambia

**DOI:** 10.2471/BLT.19.231506

**Published:** 2019-09-19

**Authors:** Cathy Green, Paula Quigley, Tendayi Kureya, Caroline Barber, Elizabeth Chizema, Haachile Moonga, Ernest Chanda, Victor Simfukwe, Bernard Mpande, Dennis Simuyuni, Kenneth Mubuyaeta, Pierre Hugo, Kim van der Weije

**Affiliations:** a Transaid, 137 Euston Road, London, NW1 2AA, England.; b DAI Global Health, London, England.; c Development Data, Lusaka, Zambia.; d National Malaria Elimination Centre, Department of Public Health, Lusaka, Zambia.; e Serenje District Health Office, Serenje, Zambia.; f MAMaZ Against Malaria, Lusaka, Zambia.; g Disacare, Lusaka, Zambia.; h Medicines for Malaria Venture, Geneva, Switzerland.

## Abstract

**Objective:**

To determine whether the administration of rectal artesunate by trained community health volunteers before referral to a health-care facility reduces the case fatality rate of severe malaria in young children in hard-to-reach communities in Zambia.

**Methods:**

We implemented a pilot project in Serenje District between July 2017 and July 2018. The project involved: (i) training community health volunteers to administer rectal artesunate to children with suspected severe malaria and refer them to a health facility; (ii) ensuring emergency transport with bicycle ambulances was available; (iii) ensuring adequate drug supplies; and (iv) ensuring health-care workers could treat severe malaria with injectable artesunate. Surveys of health facilities, volunteers and bicycle ambulance riders were performed near the beginning and end of the intervention period. In addition, data on severe malaria cases and associated deaths were obtained from health facilities and a community monitoring system.

**Findings:**

In the year before the intervention, 18 deaths occurred in 224 cases of confirmed severe malaria among children younger than 5 years seen at intervention health facilities (case fatality rate: 8%); during the intervention, 3 of 619 comparable children with severe malaria died (case fatality rate: 0.5%).

**Conclusion:**

The administration of pre-referral rectal artesunate treatment to young children with severe malaria by community health volunteers was feasible, safe and effective in hard-to-reach communities in Zambia and was associated with a substantial decrease in the case fatality rate. The project’s approach is highly adaptable and could be used in other countries with a high malaria burden.

## Résumé

**Objectif:**

Déterminer si l'administration d'artésunate par voie rectale par des agents de santé communautaires bénévoles, avant transfert vers un établissement de soins, réduit le taux de létalité du paludisme grave chez les jeunes enfants dans les communautés reculées de Zambie.

**Méthodes:**

Nous avons mis en œuvre un projet pilote dans le district de Serenje entre juillet 2017 et juillet 2018. Ce projet a impliqué de: (i) former des agents de santé communautaires bénévoles à administrer de l'artésunate par voie rectale aux enfants chez lesquels un paludisme grave était suspecté et à les transférer vers un établissement de santé; (ii) garantir la disponibilité de vélo-ambulances comme moyen de transport d'urgence; (iii) garantir des stocks suffisants de médicaments; (iv) s'assurer que les professionnels de santé puissent traiter le paludisme grave avec de l'artésunate injectable. Des enquêtes ont été effectuées auprès des établissements de santé, des bénévoles et des conducteurs de vélo-ambulances vers le début et la fin de la période d'intervention. Des données sur les cas de paludisme grave et les décès associés ont également été obtenues des établissements de santé et des systèmes de suivi communautaires.

**Résultats:**

L'année qui a précédé l'intervention, 18 décès sont survenus, sur 224 cas de paludisme grave confirmé chez des enfants de moins de 5 ans reçus dans des établissements de santé ayant participé à l'intervention (taux de létalité: 8%). Durant l'intervention, 3 enfants sont décédés, sur 619 cas comparables de paludisme grave (taux de létalité: 0,5%).

**Conclusion:**

L'administration d'un traitement pré-transfert composé d'artésunate par voie rectale chez les jeunes enfants atteints de paludisme grave, par des agents de santé communautaires bénévoles, s'est avérée réalisable, sûre et efficace dans les communautés reculées de Zambie, et était associée à une baisse conséquente du taux de létalité. L'approche adoptée dans ce projet est tout à fait adaptable et pourrait être appliquée dans d'autres pays fortement touchés par le paludisme.

## Resumen

**Objetivo:**

Determinar si la administración de artesunato rectal por parte de voluntarios sanitarios comunitarios capacitados antes de ser remitidos a un centro de atención de la salud reduce la tasa de letalidad por paludismo grave en niños pequeños de comunidades de difícil acceso en Zambia.

**Métodos:**

Se implementó un proyecto piloto en el distrito de Serenje entre julio de 2017 y julio de 2018. El proyecto involucraba: (i) capacitar a voluntarios sanitarios comunitarios para que administren artesunato rectal a niños con sospecha de paludismo grave y los remitan a un centro de salud; (ii) garantizar la disponibilidad de transporte de emergencia con bici-ambulancias; (iii) garantizar el suministro adecuado de medicamentos; y (iv) garantizar que los trabajadores de atención a la salud puedan tratar el paludismo grave con artesunato inyectable. Las encuestas a los centros de salud, voluntarios y conductores de bici-ambulancias se realizaron cerca del comienzo y el final del periodo de intervención. Además, se obtuvieron datos sobre los casos de paludismo grave y las muertes conexas de los centros de salud y de un sistema comunitario de vigilancia.

**Resultados:**

El año anterior a la intervención, se produjeron 18 muertes en 224 casos confirmados de paludismo grave entre niños menores de 5 años atendidos en los centros de salud de la intervención (tasa de letalidad: 8 %); durante la intervención, murieron 3 de los 619 niños comparables con paludismo grave (tasa de letalidad: 0,5 %).

**Conclusión:**

La administración de tratamiento con artesunato rectal previo a la remisión a niños pequeños con paludismo grave por parte de voluntarios sanitarios comunitarios fue factible, segura y eficaz en las comunidades de difícil acceso de Zambia, y se asoció a una reducción sustancial de la tasa de letalidad de los casos. El enfoque del proyecto es muy adaptable y podría utilizarse en otros países con una alta incidencia de malaria.

## ملخص

**الغرض:**

تحديد ما إذا كان إعطاء علاج الأرتيسونات عن طريق الشرج من قِبل متطوعين مدربين في مجال الصحة المجتمعية قبل الإحالة إلى مرفق للرعاية الصحية سيقلل من معدل الوفيات الناتج عن الإصابة بالملاريا الحادة لدى الأطفال الصغار في المجتمعات التي يصعب الوصول إليها في زامبيا أم لا.

**الطريقة:**

قمنا بتنفيذ مشروعًا تجريبيًا في منطقة سيرينجي في الفترة ما بين شهر يوليو/تموز لعام 2017 وشهر يوليو/تموز لعام 2018. ويتضمن المشروع ما يلي: (أولًا) تدريب متطوعين في مجال الصحة المجتمعية على إعطاء علاج الأرتيسونات عن طريق الشرج للأطفال المُشتبه في إصابتهم بالملاريا الحادة وإحالتهم إلى مرفق للرعاية الصحية؛ و(ثانيًا) ضمان توافر النقل في حالات الطوارئ بدراجات الإسعاف؛ و(ثالثًا) ضمان إمداد ما يكفي من العقاقير؛ و(رابعًا) ضمان قدرة العاملين بمجال الرعاية الصحية على معالجة الملاريا الحادة عن طريق الأرتيسونات القابلة للحقن. كما أُجريت استطلاعات حول مرافق الرعاية الصحية، والمتطوعين وراكبي دراجات الإسعاف قرب بداية فترة التدخل وانتهائها. وبالإضافة إلى ذلك، تم الحصول على بيانات بخصوص حالات الملاريا الحادة وحالات الوفيات الناتجة عنها من مرافق الرعاية الصحية ونظام المراقبة المجتمعية.

**النتائج:**

في العام السابق للتدخل، توفت 18 حالة من بين 224 حالة تم تأكيد إصابتها بالملاريا الحادة بين الأطفال الذين تقل أعمارهم عن خمس سنوات والذين تمت رؤيتهم في مرافق الرعاية الصحية للتدخل (معدل حالات الوفيات: 8%)؛ وخلال فترة التدخل، توفت 3 حالات من بين 619 طفلًا مماثلًا مصابًا بالملاريا الحادة (معدل حالات الوفيات: 0.5%).

**الاستنتاج:**

وُجد أن إعطاء علاج الأرتيسونات عن طريق الشرج للأطفال الصغار المصابين بالملاريا الحادة من قِبل متطوعين مدربين في مجال الصحة المجتمعية قبل الإحالة أمراً مجدياً وآمناً وفعالاً في المجتمعات التي يصعب الوصول إليها في زامبيا، وارتبط بانخفاض كبير في معدل حالات الوفيات. وأن نهج المشروع قابل للتكيف بدرجة كبيرة ويمكن استخدامه في بلدان أخرى ترتفع فيها نسبة الملاريا.

## 摘要

**目的:**

旨在确认转诊到医疗机构之前，由训练有素的社区卫生志愿者施用直肠青蒿琥酯能否降低赞比亚偏远社区儿童因重症疟疾的病死率。

**方法:**

我们于 2017 年 7 月至 2018 年 7 月在塞伦杰县地区开展了试点计划。该项目包括：(i) 培训社区卫生志愿者向疑似患有重症疟疾的儿童施用直肠青蒿琥酯，并将其转诊至医疗机构；(ii) 确保有救护自行车提供紧急运输；(iii) 确保充足的药品供应；以及 (iv) 确保医疗工作者能够注射青蒿琥酯以治疗重症疟疾。在临近干预期开始和结束时对医疗机构、志愿者和救护自行车骑手进行了调查。此外，我们还从医疗机构和社区监测系统获得了重症疟疾病例及其相关死亡的数据。

**结果:**

干预前一年，在干预的医疗机构发现，5 岁以下确诊患有重症疟疾的儿童有 224 例，其中 18 例死亡（病死率：8%）；在干预期间，619 例患有重症疟疾的对照组儿童中有 3 人死亡（病死率：0.5%）。

**结论:**

社区卫生志愿者在赞比亚偏远社区对转诊前的重症疟疾患儿施用直肠青蒿琥酯进行治疗可行、安全且有效，可显著降低病死率。该计划的方法适用应性强，可应用于其他疟疾负担较重的国家。

## Резюме

**Цель:**

Определение эффективности применения ректального артесуната обученными волонтерами, обеспечивающими медицинское обслуживание местного населения, до обращения в учреждение здравоохранения для снижения смертности от тяжелой малярии у детей младшего возраста в труднодоступных районах Замбии.

**Методы:**

Авторы осуществляли пилотный проект в районе Серендже в период между июлем 2017 года и июлем 2018 года. Проект подразумевал: (i) обучение добровольцев, осуществляющих медицинское обслуживание местного населения, применению ректального артесуната у детей с подозрением на тяжелую малярию и направление таких детей в больницу; (ii) обеспечение транспорта для неотложной перевозки больных, в том числе велосипедов скорой помощи; (iii) обеспечение достаточного запаса лекарств; (iv) обеспечение возможности лечения тяжелых случаев малярии посредством инъекции артесуната, выполняемой обученными добровольцами. Перед началом и по окончании мер вмешательства проводился опрос больниц, добровольцев и велосипедистов скорой помощи. Кроме того, из больниц и систем мониторинга общин были получены данные о случаях тяжелой малярии и связанной с ними смертностью.

**Результаты:**

В предшествовавший вмешательству год от подтвержденной тяжелой малярии умерло 18 детей в возрасте до 5 лет из 224 заболевших и принятых в учреждения здравоохранения (уровень смертности 8%); в ходе вмешательства из 619 детей со сравнимой тяжестью малярии умерло 3 (уровень смертности 0,5%).

**Вывод:**

Применение волонтерами, обеспечивающими медицинское обслуживание местного населения, ректального артесуната для лечения тяжелой формы малярии у маленьких детей, проживающих в труднодоступных общинах Замбии, до направления к врачу оказалось осуществимым, безопасным и эффективным и было связано со значительным снижением уровня смертности. Подход, реализованный в проекте, допускает высокую степень адаптации и может использоваться в других странах с высоким бременем малярии.

## Introduction

Despite an estimated 60% reduction in deaths due to malaria over the past two decades, global progress in combatting the disease has stalled recently, in some countries, previous mortality gains have even been reversed. In 2017, 61% of the 435 000 malaria deaths worldwide were among children younger than 5 years.^1^ The World Health Organization (WHO) issued a call to action and asked countries with a high malaria burden to develop local strategies for areas of greatest need, with a focus on strong primary health care.^2^

Among patients with severe malaria who fail to reach a health facility, an estimated 90% will die.^3^ One intervention that could drastically reduce child deaths due to severe malaria is administering rectal artesunate suppositories before referral to a health facility. This approach buys vital time for severely ill children who live far from a facility that can provide treatment, ideally with injectable artesunate. Although the efficacy and safety of rectal artesunate has been confirmed,^4^^,^^5^ little is known about its use at the community level.

In Zambia, many household and community barriers prevent the timely use of malaria services and access to the most effective medicines.^6^^–^^8^ In 2017, an estimated 7618 malaria deaths occurred in the country, many among young children.^1^ In April 2017, the health ministry announced through the National Malaria Elimination Centre that the country was committed to eliminating malaria by 2021.^9^ Although rectal artesunate was included in national malaria guidelines,^10^ this treatment was not available anywhere in the country at that time. Moreover, injectable artesunate, as recommended by WHO since 2011 for treating severe malaria at referral facilities, was not available below district hospital level.

In Serenje District in Zambia’s Central Province, the incidence of malaria in children younger than 5 years was estimated to be 490 per 1000 between 2013 and 2015,^11^ with *Plasmodium falciparum* accounting for most cases. Before 2017, many people with severe malaria failed to reach a health facility. Prompt action was prevented by limited awareness of the signs and symptoms of severe malaria, the expenses incurred when seeking treatment, long distances to health facilities (up to 80 km) and a lack of suitable transport. Several interventions to improve community engagement have been implemented in Serenje. Two interventions, Mobilising Access to Maternal Health Services in Zambia between 2010 and 2013 and a successor project between 2014 and 2016, involved training community health volunteers to address barriers to, and delays in, the timely uptake of essential maternal and newborn health services.^12^^–^^14^

In July 2017, a collaborative project, MAMaZ Against Malaria, was established to build on these foundations. The project involved a 12-month pilot study to test the feasibility of introducing pre-referral treatment with rectal artesunate in hard-to-reach communities. The main objective was to reduce the case fatality rate of severe malaria in children 6 months to 6 years of age by: (i) ensuring the availability of drugs for severe malaria in communities and health facilities; (ii) training community health volunteers to administer rectal artesunate, and to promptly refer and follow up cases; (iii) improving health-care workers’ capacity to manage cases; and (iv) building the government’s capacity to integrate this intervention into routine activities. The aims of this study were to determine whether the project reduced the severe malaria case fatality rate in young children and to evaluate the feasibility of administering rectal artesunate suppositories in hard-to-reach communities before referral to a health facility.

## Methods

We performed an observational, cross-sectional study using data from three sources: (i) surveys carried out near the beginning and end of the intervention period; (ii) health facilities, which contributed data on malaria to the Zambian Health Management Information System; and (iii) a community monitoring system. In addition, qualitative data collected included: (i) case studies; (ii) feedback from government officials; and (iii) reports of informal discussions with community health volunteers and communities. The project was implemented in partnership with the National Malaria Elimination Centre and the district health management team.

We selected Serenje District because earlier projects showed good results and the district was highly involved. The choice was also influenced by the modest budget of 300 000 United States dollars (US$) and the short duration of the project. We selected eight of the 24 health facilities in Serenje as intervention sites: five rural health centres (i.e. Kabamba, Kabundi, Malcolm Moffat, Mulilima and Serenje Urban) and three rural health posts (i.e. Kashishi, Kalela and Muzumani). All had participated in two earlier projects. Together, these facilities included 45 communities in their catchment areas and covered a population of 54 000 (i.e. 40% of the district population). The intervention was implemented between July 2017 and July 2018. A detailed design mission was undertaken in July 2017 and baseline and endline surveys were carried out in August 2017 and May 2018, respectively.^15^

We developed a training module on rectal artesunate that covered other child health issues (i.e. uncomplicated malaria, pneumonia and severe diarrhoea) and the training used a cascade strategy.^16^ Community health volunteers preferentially included volunteers from Safe Motherhood Action Groups (a government maternal health initiative) who had participated in earlier projects and volunteers trained in Integrated Community Case Management. Volunteers were trained to administer rectal artesunate to children showing signs of severe malaria, that is, fever and at least one of the following signs: fitting; lethargy or unconsciousness; inability to eat or drink; and vomiting everything, and to refer them promptly to a designated health facility where they could receive treatment.^2^ An additional group of volunteers (all Safe Motherhood Action Group volunteers) was trained only in community mobilization: awareness of the danger signs of severe malaria and of the need for prompt action was increased through community discussion groups.

Training emphasized social inclusion and reaching vulnerable and marginalized women and their children.^12^^,^^17^ To address affordability and other practical constraints that prevented prompt responses to health emergencies, community-managed food banks and emergency savings schemes were revitalised or established. In addition, the emergency transport system was strengthened by: (i) training the riders of bicycle ambulances, which have trailers; (ii) repairing existing bicycle ambulances or purchasing new ones; and (iii) placing bicycle ambulances in sites where there was no alternative transport. Bicycle ambulances were placed in the catchment areas of five of the eight intervention health facilities. The community monitoring system (which covered counter-referral) was adapted to accommodate the new focus on severe malaria.

Pre-referral rectal artesunate was made available in all project areas. The national regulatory authority and procurement agency ensured the drug was registered for future national procurement. When a community health volunteer observed the signs of severe malaria, the individual was given a rapid diagnostic test for malaria. If infection was confirmed, the volunteer immediately administered rectal artesunate, in line with WHO recommendations.^18^

Health-care workers at facilities underwent refresher training to improve severe malaria case management, including the use of injectable artesunate. The district health management team and National Malaria Elimination Centre ensured that injectable artesunate was available at all facilities. We encouraged health-care workers to support and supervise community health volunteers and bicycle ambulance riders during monthly community outreach sessions. We trained community health volunteers to follow up patients and look for potential treatment side-effects. All preparatory and training activities were completed before the main malaria transmission season started in November 2017.

### Data collection

The principal outcome indicators were: (i) the proportion of young children with suspected severe malaria who received pre-referral rectal artesunate in the community; (ii) the proportion of these children who were then referred to a health facility; and (iii) the proportion who also received a detailed counter-referral form. We developed two assessment tools: (i) a health facility audit tool, which captured information on personnel, commodities and severe malaria case management practices; and (ii) a structured interview tool, which was used with community health volunteers and bicycle ambulance riders. Fieldwork was conducted by independent enumerators who collected data using tablet computers and computer-assisted interview software. Interview respondents were not remunerated. Staff members at health facilities recorded eight severe malaria-related indicators each month, the data were checked against facility registers during baseline and endline surveys. Community health volunteers compiled data on the number of children who were seen, tested for malaria, referred to a facility, used a bicycle ambulance and benefitted from community support. Project staff assured the quality of these data each month. All data sources were used to ensure results were valid. In addition, the district health management team monitored severe malaria case management practices during routine supervisory visits to health facilities.

As the Zambian Health Management Information System, which collects data from health facilities, categorizes children as younger than 1 year and1 to younger than 5 years of age, these age groups combined were used as a proxy for the intervention’s target age group of 6 months to 6 years of age. We compared facility data from July 2016 to June 2017 and from November 2017 to March 2018, which covered two consecutive malaria peak seasons. Data on outcome indicators in children aged 6 months to 6 years were available from community health volunteers each month and from baseline and endline surveys. During the surveys, a sample of approximately 50% of all trained community health volunteers was interviewed: 300 in the baseline survey and 277 in the endline survey.

The research protocol was approved in August 2017 by the independent ERES Converge ethical review board in Zambia and the National Health Research Authority. Consent on use of the information and photographs was obtained from all participants.

## Results

In total, 543 community health volunteers underwent training of some form: (i) 180 Safe Motherhood Action Group volunteers and 45 volunteers trained in Integrated Community Case Management and other malaria volunteers in administering rectal artesunate; (ii) 252 volunteers in community mobilization for severe malaria; and (iii) 66 bicycle ambulance riders in emergency transport, riders were encouraged to train two or three additional community members.

At the baseline survey in August 2017, no injectable or rectal artesunate (for community distribution) was available at intervention health facilities. During the project, 3000 units of quality assured, rectal artesunate were supplied and the district was helped to obtain injectable artesunate from the national medical store. By the endline survey in May 2018, all facilities except one had enough drug supplies to last at least 30 days and there were no stock-outs of commodities for severe malaria (e.g. gloves and rapid diagnostic tests). Each trained health-care worker managed at least one case of severe malaria. All intervention health facilities found a way to supervise community health volunteers, whether during facility meetings or outreach sessions for caregivers of children younger than 5 years of age in the community, which helped sustain motivation.

The community health volunteers identified 1215 children 6 months to 6 years of age with suspected severe malaria. All 1215 were administered rectal artesunate and provided with referral forms to a health facility. Subsequently, 72% (875/1215) received counter-referral forms from the facilities. Knowledge of the danger signs of severe malaria in the community improved significantly: at the endline survey, 85% (235/277) of community health volunteers knew three or more signs compared with 50% (150/300) at the baseline survey (*P* < 0.05).

At the baseline survey, community health volunteers reported identifying an average of 0.4 cases per month of uncomplicated or severe malaria among children in the preceding 12 months. At the endline survey, the average was 3.5 cases per month (*P* < 0.05), almost nine times greater. Community monitoring system data confirmed this finding: together community health volunteers and bicycle ambulance riders reported 11 638 malaria cases among children between November 2017 and May 2018, which was equivalent to 3.14 cases per volunteer per month. Overall, 71% (714/1007) of suspected severe malaria cases in children that occurred in areas with emergency transport were transferred to a health facility by bicycle ambulance. Bicycle ambulances also carried 838 children who had another health emergency during the project period. By May 2018, each rider had transported an average of 10 children since their training in November 2017.

A verification exercise carried out in June 2018 found that 19 of 27 (70%) children surveyed who had received rectal artesunate were diagnosed with severe malaria at a health facility. No treatment side-effects were observed during follow-up.

**Fig. 1:**
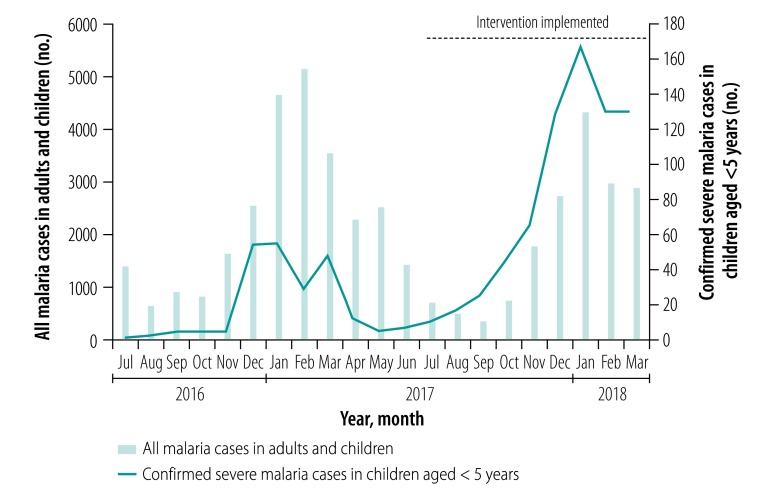
Number of people with malaria, Serenje District, Zambia, 2016–2018

Note: Data were obtained from the Zambian Health Management Information System, which recorded information from the eight health facilities participating in the intervention.

The total number of all malaria cases in adults and children recorded through the Zambian Health Management Information System at intervention health facilities during the peak malaria season (i.e. November to March) was 16% lower during the project than in the preceding year (i.e. 14 686 versus 17 529, respectively). However, the number of cases reported in children younger than 1 year of age increased by 15% (i.e. from 1434 to 1651) during this period (Fig. 1 and Table 1), which suggests that identification and referral rates rose in this age group. Between July 2016 and June 2017, the year before the start of the project, 18 deaths resulted from 224 cases of confirmed severe malaria among children younger than 5 years of age who presented at the eight intervention health facilities, giving a case fatality rate of 8% (18/224). Fatalities in the community were excluded. Between November 2017 and March 2018 (which covers the malaria peak season), 619 confirmed cases of severe malaria in children younger than 5 years were recorded at intervention health facilities (Table 1) and three deaths occurred, including one of a child who came from outside the project area. The corresponding case fatality rate was 0.5% (3/619). Consequently, 15 fewer malaria-related deaths were recorded among children with confirmed severe malaria at intervention health facilities during the project compared with the previous 12 months, despite the number with confirmed severe malaria increasing from 224 to 619.

**Table 1:** Malaria cases and deaths at intervention sites,^a^ community intervention to reduce childhood deaths from severe malaria, Serenje District, Zambia, 2016–2018

Period	No. all malaria cases				No. confirmed severe malaria cases			No. deaths attributed to severe malaria			No. with side-effects from malaria treatment	
	Adults and children	Children aged < 1 year	Children aged 1 to < 5 years		Children aged < 1 year	Children aged 1 to < 5 years		Children aged < 1 year	Children aged 1 to < 5 years		Children aged < 1 year	Children aged 1 to < 5 years
**2016**												
July	1 388	81	185		0	1		0	0		0	0
August	617	34	76		2	0		0	1		0	0
September	889	59	83		0	4		0	0		1	0
October	823	112	150		0	4		0	0		0	0
November	1 639	126	369		0	4		0	0		0	0
December	2 550	354	1 127		3	51		3	0		0	0
**2017**												
January	4 649	236	1 346		10	45		2	1		0	0
February	5 141	383	1 991		6	23		4	0		0	1
March	3 550	335	1 333		15	33		2	2		0	0
April	2 285	117	855		2	10		1	2		0	0
May	2 509	174	813		0	5		0	0		0	0
June	1 408	85	420		4	2		0	0		0	0
July	697	62	183		3	7		0	0		0	0
August	500	42	144		0	16		0	0		0	0
September	335	29	115		4	21		0	0		0	0
October	736	60	314		13	31		0	1		0	0
November	1 768	150	683		13	52		0	0		0	0
December	2 725	249	929		30	97		0	0		0	0
**2018**												
January	4 324	590	1 427		60	107		0	2		0	0
February	2 974	319	966		37	93		0	0		0	0
March	2 895	343	906		49	81		0	1		0	0
**November 2016–March 2017^b^**	17 529	1 434	6 166		34	156		11	3		0	1
**November 2017–March 2018^b^**	14 686	1 651	4 911		189	430		0	3		0	0
**Net change between two periods**	–2 843	+217	–1 255		+155	+274		–11	0		0	–1

^a^ Data were obtained from the Zambian Health Management Information System, which recorded information from the eight health facilities participating in the intervention.

^b^ The peak malaria season in Serenje District lasts from November to March.

## Discussion

The intervention achieved its two intended outcomes: all children with suspected severe malaria in the community were administered rectal artesunate; and all were referred to a health facility for treatment. A verification exercise found that severe malaria was confirmed in 70% of children studied. The other 30% were diagnosed with uncomplicated malaria. Community health volunteers were judged to be administering rectal artesunate responsibly and were correct to err on the side of caution by making sure all suspected cases reached a health facility. A larger, more detailed verification exercise would determine whether rectal artesunate was overprescribed or injectable artesunate was underprescribed. Anecdotal evidence indicated that the latter was more likely. The success of the project encouraged the district health management team to make injectable artesunate available at rural health facilities and increased demand for severe malaria services helped ensure the drug was widely available. Participating health facilities reported greater drug availability and an improved continuum of care from the community up through the referral system.

The intervention was associated with a reduction in the severe malaria case fatality rate. However, fatality rates at the baseline and endline surveys were not directly comparable because rectal artesunate treatment was not available in the community before the intervention and rural health facilities were using other medications to treat severe malaria or referring cases to the district hospital, where injectable artesunate was available. Nevertheless, the data available indicate that the intervention was highly likely to have improved severe malaria outcomes. Subsequent research should include both intervention and control sites.

Potential confounders that may have affected malaria mortality include other measures used to prevent or treat the disease. For example, indoor residual spraying and insecticide-treated nets were common across the district. Insufficient funds were available to conduct a case–control or population survey. However, government stakeholders involved in the project provided anecdotal evidence on the relative contribution of rectal and injectable artesunate in intervention sites compared with other locations.

The project used an “end-to-end approach” in tackling severe malaria in hard-to-reach communities. First, a technological innovation (i.e. pre-referral rectal artesunate) was grounded in community engagement, which reduced barriers to, and delays in, health service utilization. Second, emergency transport systems connecting communities to health facilities were strengthened. Third, health-care workers were trained in severe malaria case management, and in supervising and supporting community health volunteers. Fourth, innovative teaching and learning methods appropriate for a low literacy context were used to train community health volunteers. Consequently, volunteers quickly acquired knowledge, retained that knowledge, and rapidly developed the capacity to train and mobilize others.^15^ Fifth, the entire community was involved, which encouraged wide social acceptance of behavioural change. Maternal health projects in Zambia and northern Nigeria used similar community health volunteer training, and community mobilization approaches to improve maternal and child health and immunization levels, with positive results.^12^^,^^19^

The community health volunteers came from a variety of backgrounds. Although most were Safe Motherhood Action Group volunteers, we observed no substantial difference in performance between these volunteers and those who had prior malaria training. In scaling up pre-referral rectal artesunate treatment across the country, the health ministry is encouraged to consider training all available community health volunteers at the community level because this approach is consistent with integrated primary health care and promotes universal health coverage. During our project, the number of malaria-trained community health volunteers increased from the government target of 1 per 500 people to 1 per 250. This ratio helped increase access to health care, improved the identification of severe malaria cases and broadened coverage of pre-referral rectal artesunate. The large number of community health volunteers trained provided opportunities to share workloads and improved motivation, which are helpful for volunteer retention and the sustainability of community health initiatives. However, cost needs to be considered when the intervention is extended. In a district population of 100 000, we estimated it would cost US$ 22 400 to provide district-wide coverage based on a ratio of one community health volunteer per 250 people, which should be affordable for governments, considering the likely benefits.

The project demonstrated that adopting a comprehensive health systems approach was important for reducing severe malaria mortality in rural Zambia and that embedding the intervention in government structures was key to ensuring sustainability. The essential components were a reliable supply of quality assured drugs; training for community health volunteers; community engagement; an emergency transport system; training for health-care workers; and supportive supervision for health-care workers and community health volunteers. However, given the project’s modest project budget, we were unable to identify the components most critical to success. Efforts to eliminate malaria in Zambia will require a similar comprehensive approach, with interventions needed on both the supply and demand sides of health care. 

In conclusion, pre-referral rectal artesunate treatment is intended for use in areas where access to health care is poor and patients’ lives may be threatened without an intervention. Our pilot project demonstrated that training community health volunteers to administer rectal artesunate to children with suspected severe malaria in hard-to-reach rural areas of Zambia was feasible, safe and effective, and confirmed the pre-treatment’s potential in the fight against malaria. This approach is highly adaptable and could be used in other countries with a high malaria burden, both within and outside sub-Saharan Africa. The evidence obtained and the lessons learnt are being applied in a successor project that is supporting the health ministry’s preparations for national implementation.
